# Clinical significance and immune microenvironment association of cuproptosis‐related genes in pan‐cancer

**DOI:** 10.1113/EP092310

**Published:** 2025-06-25

**Authors:** Xinyu Ge, Kaijing Wang, Tian Zhao, Jinyi Wang, Jie Liu, Zhengliang Sun, Zhengjun Chai, Wen Zhang, Chengbao Li, Yan Xu, Guohan Chen

**Affiliations:** ^1^ Department of Thoracic Surgery, Shanghai East Hospital Tongji University School of Medicine Shanghai China; ^2^ Department of Hepatological Surgery, General Surgery, Shanghai East Hospital Tongji University School of Medicine Shanghai China; ^3^ Department of Anesthesiology Shandong Provincial Hospital Affiliated to Shandong First Medical University Jinan Shandong China; ^4^ Department of Pathology, Shanghai East Hospital Tongji University School of Medicine Shanghai China

**Keywords:** cuproptosis‐related genes, pan‐cancer, prognosis, tumour immunity microenvironment

## Abstract

Recent studies highlight the important roles of cuproptosis in cancer cells. However, the roles of the cuproptosis‐related genes (CRGs) in different cancers are still not fully understood. Comprehensive analysis was performed using open‐source bioinformatic platforms to disclose the expression profiles, prognostic significance, genomic and epigenetic characteristics, immune microenvironment, and drug sensitivity of CRGs including *FDX1*, *LIAS*, *LIPT1*, *DLD*, *DLAT*, *PDHA1*, *PDHB*, *SLC31A1*, *MTF1*, *GLS*, *CDKN2A*, *HSPA4* and *ATP7B*. The colon cancer samples were further obtained to verify the correlation between *CDKN2A* expression and immune cell infiltration by fluorescence staining. We demonstrated the expression level, methylation and the copy number variant feature, as well as the prognostic significance of CRGs in pan‐cancers. The expression of CRGs, especially *PDHB*, *LITP1*, *ATP7B*, *HSPA4* and *CDKN2A*, was significantly correlated with pathological stages. The genetic alteration of CRGs was explored, and *CDKN2A* was the most frequently altered gene with alteration rate as high as 17% in 10,953 tumour patients. In addition, we revealed a relationship to the tumour immune microenvironment (TIME) and drug resistance in pan‐cancer. Moreover, *CDKN2A*, which was closely correlated to pan‐cancer prognosis, was especially analysed including TIME alteration, genomic heterogeneity and tumour stemness. Fluorescence images of colon cancer from different patients demonstrated a positive correlation between *CDKN2A* expression and the number of CD45^+^ immune cells. Our research has provided a comprehensive understanding of cuproptosis regulators and revealed potential prognostic biomarkers and therapeutic targets for cancers.

## Abstract (highlights)

- **What is the central question of this study?**
  What are the genomic and epigenetic characteristics of cuproptosis‐related genes (CRGs) in different cancers?
- **What is the main finding and its importance?**
  The present study disclosed the expression profiles, prognostic significance, immune microenvironment and drug sensitivity of 13 CRGs including *FDX1*, *LIAS*, *LIPT1*, *DLD*, *DLAT*, *PDHA1*, *PDHB*, *SLC31A1*, *MTF1*, *GLS*, *CDKN2A*, *HSPA4* and *ATP7B*. *CDKN2A* was specially analysed including tumour immune microenvironment alteration, genomic heterogeneity and tumour stemness. This research provided a comprehensive understanding of cuproptosis regulators and revealed potential prognostic biomarkers and cancer therapeutic targets.

## 1 INTRODUCTION

Cancer is one of the leading causes of death and brings heavy burdens all over the world. In 2018, there were approximately 18.1 million new cancer cases and 9.6 million cancer deaths (Bray et al., 2018). However, the underlying molecular mechanism and potential therapeutic targets remain poorly understood.

The tumour immune microenvironment (TIME) has been highlighted to play a vital role in tumour progression and to influence patient prognosis (Bruni et al., 2020). Anticancer immunotherapy, especially immune checkpoint inhibitors (ICIs), has brought new hope for cancer management (Galluzzi et al., 2020; Tu et al., 2022). However, drug resistance (Huang & Zappasodi, 2022) and toxicity (Waldman et al., 2020) are still waiting to be solved. Comprehensive analysis of the TIME will help to reveal the mechanisms of immunotherapy response and resistance, leading to improved clinical outcomes and the development of new therapeutic strategies.

Copper is an essential micronutrient involved in a variety of basic biological functions (Svetlana et al., 2024). Cancer cells have a high demand for copper, and copper concentrations in tumours have repeatedly been found to be elevated (Shanbhag et al., 2021). Recent studies have begun to explore the specific effect of copper on the oncogenic mechanism and anti‐neoplastic drug resistance. The anti‐neoplastic and anti‐metastatic benefits of copper depletion have been shown in various animal and human researches (Brewer, 2014; Lopez et al., 2019). As a newly identified copper‐dependent form of cell death, cuproptosis is distinct from all other known ones and has been demonstrated to play a vital role in tumour development (Tang et al., 2024; Tsvetkov et al., 2022). As shown in a recent study, the copper ionophore elesclomol binds to and transports Cu(II) into mitochondria, where Cu(II) is reduced to Cu(I) by ferredoxin 1 (FDX1). This activity of elesclomol promotes the cytotoxic effects of copper, and lipophobic antioxidants and apoptosis inhibitors fail to rescue this copper‐induced decrease in the viability of several human lung cancer cell lines (Tsvetkov et al., 2022). Cuproptosis is strongly connected to the modulation of antitumour immunity. Thus, a thorough recognition of the mechanisms involved in the modulation of copper metabolism and cuproptosis may facilitate improvement in cancer management. The field of cuproptosis is nascent in many ways and thus, further studies are urgently needed to reveal the molecular mechanisms and clinical relevance of different cancers.

In the current study, we conducted a comprehensive analysis based on various databases. We visualized the expression profiles of cuproptosis‐related genes (CRGs) and explored their prognostic significance, as well as their relationship to TIME and drug resistance in pan‐cancer. Moreover, *CDKN2A*, which was closely correlated to pan‐cancer prognosis, was especially analysed, including TIME alteration, genomic heterogeneity and tumour stemness. Our research may provide additional evidence for prognostic biomarkers and therapeutic targets for cancer.

## 2 METHODS

### 2.1 Ethical approval, data retrieval and preprocessing

Colon cancer specimens were obtained from patients referred to Shanghai East Hospital for surgical management. Written informed consent was obtained from the participants. The present study conformed to the standards set by the latest revision of the *Declaration of Helsinki* and was approved by the institutional review board of Shanghai East Hospital (2019tjdx39). Tumour tissue and normal tissue RNA seq data in the TPM format of 33 human cancers in the Cancer Genome Atlas (TCGA) and Genotype‐Tissue Expression (GTEx) databases and clinical information were downloaded from the UCSC Xena data portal (https://xenabrowser.net/datapages/). The expression data were normalized to transcripts per kilobase million (TPM) values before further analysis. The abbreviations for various cancer types are listed in Supporting information, Table S1.

### 2.2 The expression of CRGs in human cancers

Tumour tissue and normal tissue RNA seq data from TCGA and GTEx databases were used to compare the differential expression of CRGs in various types of tumours using the online platform for gene expression profiling interactive analysis (GEPIA2, http://gepia2.cancer‐pku.cn/#index; Tang et al., 2019). We obtained the correlation between each CRG expression and the pathological stage of pan‐cancer by the ‘stage plot’ module. We also explored the differential expression of *PDHB* in each cancer using the ‘box plot’ module. TNMplotter (https://tnmplot.com/analysis/) was utilized to study the differential expression of *PDHB* in tumour, normal and metastatic tissues across human cancers (Bartha & Győrffy, 2021). The Human Protein Atlas (HPA) database (www.proteinatlas.org) was used to show the protein level of CDKN2A via immunohistochemistry staining.

### 2.3 Survival prognosis analysis

The relationship between CRGs and patient survival in different cancers was analysed using GEPIA2 based on TCGA and the GTEx data. The median was selected as the group cutoff. An overall survival (OS) map and Kaplan–Meier plot comparing high and low expression of CRGs in pan‐cancer were obtained. Especially, the prognostic values of *CDKN2A* and *PDHB* on the subtype of indicated tumour were also investigated using GEPIA2.

### 2.4 Genetic alteration of CRGs

The online tool cBioPortal (https://www.cbioportal.org/) integrating genetic alteration data was used to analyse the genetic alteration of CRGs in human tumours (Cerami et al., 2012; Gao et al., 2013). The alteration landscape of CRGs in pan‐cancer was depicted based on TCGA Pan‐Cancer *Atlas* studies. An overall survival comparison was performed between CRG‐altered and unaltered individuals. In particular, the clinical features and prognoses of *CDKN2A*‐altered and unaltered individuals were analysed at the pan‐cancer level.

### 2.5 The methylation landscape of CRGs

Illumina human methylation 450k level 3 data were downloaded from the TCGA database. The CRG mRNA expression data and methylation data were merged by TCGA barcode. Spearman correlation analysis was conducted to determine the correlation between gene mRNA expression and the methylation level. A total of 14 cancer types (including thyroid carcinoma (THCA), kidney renal papillary cell carcinoma (KIRP), bladder urothelial carcinoma (BLCA), liver hepatocellular carcinoma (LIHC), head and neck squamous cell carcinoma (HNSC), breast invasive carcinoma (BRCA), lung adenocarcinoma (LUAD), prostate adenocarcinoma (PRAD), oesophageal carcinoma (ESCA), kidney chromophobe (KICH), lung squamous cell carcinoma (LUSC), kidney renal clear cell carcinoma (KIRC), stomach adenocarcinoma (STAD), colon adenocarcinoma (COAD)), which have more than 10 paired tumour and adjacent non‐tumour samples, were selected to perform the differential analysis of methylation between tumour and normal sample groups. Median methylation level was used to divide tumour samples into high and low methylation groups. R package survival was applied to fit survival time and survival status within two groups. The Cox proportional‐hazards model was constructed to obtain the risk ratio (hazard ratio) of the high methylation group compared with the low methylation group.

### 2.6 Copy number variation of CRGs

The copy number variation (CNV) data of 11,495 samples downloaded from the TCGA database were processed through GISTIC2.0 to identify significantly altered regions of amplification or deletion across sets of patients. The CRG expression data and CNV raw data were merged by TCGA barcode. Spearman correlation analysis was conducted to obtain the correlation between gene expression and CNV. To explore the survival differences between CNV and the wide type of each gene, CNV data and clinical survival data were merged by sample barcode. R package survival was used to fit survival time and survival status within groups.

### 2.7 Gene set variation analysis score and immune infiltration

The association between immune cell infiltrates and CRG expression level was estimated with the Gene Set Cancer Analysis database (GSCA, http://bioinfo.life.hust.edu.cn/GSCA/), an integrated platform for genomic, pharmacogenomic and immunogenomic gene set cancer analysis. The infiltrates of 24 immune cells were evaluated through ImmuCellAI. The R package GSVA was applied to calculate the gene set variation analysis (GSVA) score of CRGs. The association between immune cell infiltrates and CRG expression was represented by a correlation coefficient through Spearman correlation analysis.

### 2.8 Correlation analysis between CDKN2A and immunomodulators, immune checkpoint, immune cell infiltration, genomic heterogeneity and stemness scores

The normalized pan‐cancer sets including TCGA, TARGET, and GTEx (PANCAN, *N* = 19,131, *G* = 60,499) were downloaded from the UCSC database (https://xenabrowser.net/). We extracted the gene expression profiles for each tumour and calculated the stromal score of the tumour in each patient using the R package ESTIMATE (Version 1.0.13). The infiltration scores of B cells, T cell CD4, T cell CD8, neutrophils, macrophages and dendritic cells in each patient were evaluated according to gene expression using the R packages IOBR (version 0.99.9) of the Timer method (Li et al., 2020). We calculated the tumour mutational burden (TMB) of each tumour using the TMB function of the R software package maftools (Version 2.8.05). The microsatellite instability (MSI) score, neoantigen (NEO), and homologous recombination deficiency (HRD) data of each tumour were obtained based on previous studies (Bonneville et al., 2017; Thorsson et al., 2018). Six stemness scores including RNAss, EREG.EXPss, DNAss, EREG‐METHss, DMPss and ENHss were calculated based on a previous study (Malta, Gevaert, et al., 2018). The tumours with less than three samples were eliminated. Log2 (*x* + 0.001) transformation was performed for each expression value and a Pearson correlation was performed for all the correlation analyses using the corr.test function from the R package Psych (Version 2.1.6).

### 2.9 Immunofluorescence staining

Colon cancer tissues were fixed in 4% paraformaldehyde and then dehydrated and embedded in paraffin. Tumour sections were incubated with anti‐CD45 (1:100, ab4076, Abcam, Waltham, MA, USA) and anti‐CDKN2A antibodies (1:100, cat. no. 18769, Cell Signalling Technology, Danvers, MA, USA) at 4°C overnight. After washing three times with phosphate‐buffered saline, fluorescein‐isothiocyanate‐conjugated secondary antibodies (1:1000, ab150077, Abcam; and 1:1,000, cat. no. 8889, Cell Signalling Technology) were incubated at room temperature in the dark for 1 h. After 4′,6‐diamidino‐2‐phenylindole (cat. no. MBD0020, Sigma‐Aldrich, St Louis, MO, USA) staining, fluorescence images were captured under the fluorescence microscope (Leica, Wetzlar, Germany).

### 2.10 Drug sensitivity and CRG expression

A correlation between drug sensitivity and the CRGs was performed using the Gene Set Cancer Analysis (GSCA) database. Briefly, the IC_50_ of small molecules and the corresponding mRNA gene expression from Genomics of Drug Sensitivity in Cancer (GDSC) and Genomics of Therapeutics Response Portal (CTRP) were collected. Pearson correlation analysis was performed to obtain the correlation between gene mRNA expression and drug IC_50_. The drugs were ranked by the integrated level of correlation coefficient and false discovery rate (FDR) of the selected genes. A bubble plot of the top 30 ranked drugs was drawn to summarize the correlations between the CRGs and drugs. The blue bubbles represent negative correlations and the red bubbles represent positive correlations. Bubble size was positively correlated with the FDR significance. The black outline border indicated FDR ≤ 0.05.

### 2.11 Statistical analysis

Data analysis was performed with the R (version 4.0.5, R Foundation for Statistical Computing, Vienna, Austria) and R Bioconductor packages. The comparison between the two groups was analysed using the Wilcoxon test. The comparison of three or more groups was analysed using the Kruskal–Wallis test. The Kaplan–Meier method was utilized to generate survival curves using the ‘Survminer’ R package. The differences in OS and progression‐free survival (PFS) between groups were determined using a log‐rank test. Pearson's correlation coefficient was used in all correlation analyses. A *P*‐value <0.05 was considered statistically significant.

## 3 RESULTS

### 3.1 Expression pattern of CRGs in pan‐cancer

**FIGURE 1:**
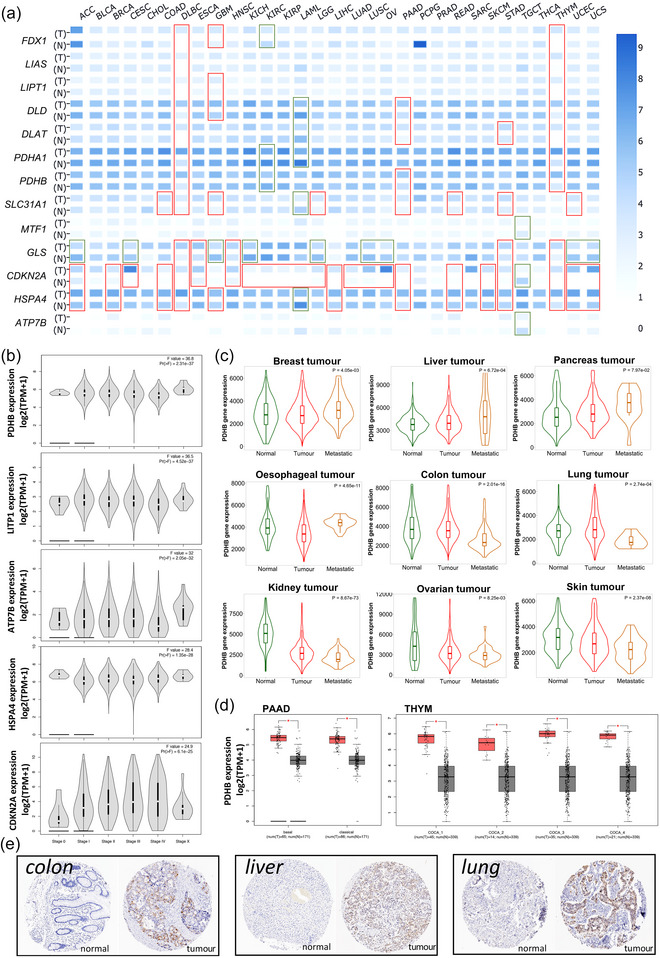
The expression pattern of CRGs in various cancers. (a) The expression map of CRGs in 31 tumour types based on the TCGA and GTEx databases (T, tumour; N, normal; red box for upward, green box for downward). (b) The expression of CRGs in different pathological stages of pan‐cancer. (c) The differential expression of *PDHB* in normal, tumour and metastatic tissues across different human cancers. (d) The differential expression of *PDHB* in the subtypes of PAAD and THYM. (e) Immunohistochemistry staining showing the expression of CDKN2A protein in the indicated tumour and normal tissues based on the HPA database. ^*^ *P *< 0.01.

We first explored the expression difference of 13 CRGs using TCGA data and found that CRGs were differentially expressed between the 14 tumour tissues and adjacent normal tissues (Supporting information, Figure S1). Due to the lack of normal control of several tumours in the TCGA database, we also obtained an expression map of CRGs in 31 tumour types based on the TCGA and GTEx databases. As shown in Figure 1a, CRGs were differentially expressed in multiple tumour types, especially *GLS*, which was down‐regulated in most of the tumours, as well as *CDKN2A* and *HSP4A*, which were widely overexpressed in the tumour tissues. In addition, we found differential expression of most CRGs in several tumours including diffuse large B‐cell lymphoma (DLBC), glioblastoma multiforme (GBM), pancreatic adenocarcinoma (PAAD) and thymoma (THYM), which implied a vital role of CRGs in these tumours. The CRG expression in different pathological stages of pan‐cancer was explored, and the results showed that the expression of CRGs was significantly correlated with pathological stages. The top five significantly differentially expressed CRGs, namely *PDHB*, *LITP1*, *ATP7B*, *HSPA4* and *CDKN2A*, are shown in Figure 1b. Further, *PDHB* expression in the metastatic tumour of the breast, liver, pancreas and oesophagus was significantly higher than in the primary tumour. The expression of *PDHB* was lower in the metastatic tumour of the colon, lung, kidney, ovarian and skin than in the primary tumours (Figure 1c). We also found differential expression of PDHB in the subtype of PAAD and THYM (Figure 1d). Given the general differential expression of *CDKN2A* in pan‐cancer, CDKN2A protein level in the tumour and normal tissues was explored based on the HPA database. Immunohistochemistry showed a higher level of CDKN2A protein expressed in the tumour tissue of the colon, liver and lung than in normal tissues (Figure 1e). These results presented a different expression pattern of CRGs between tumour and normal tissues and implied a potential role of the CRGs in tumour progression.

### 3.2 The prognostic value of CRG expression

**FIGURE 2:**
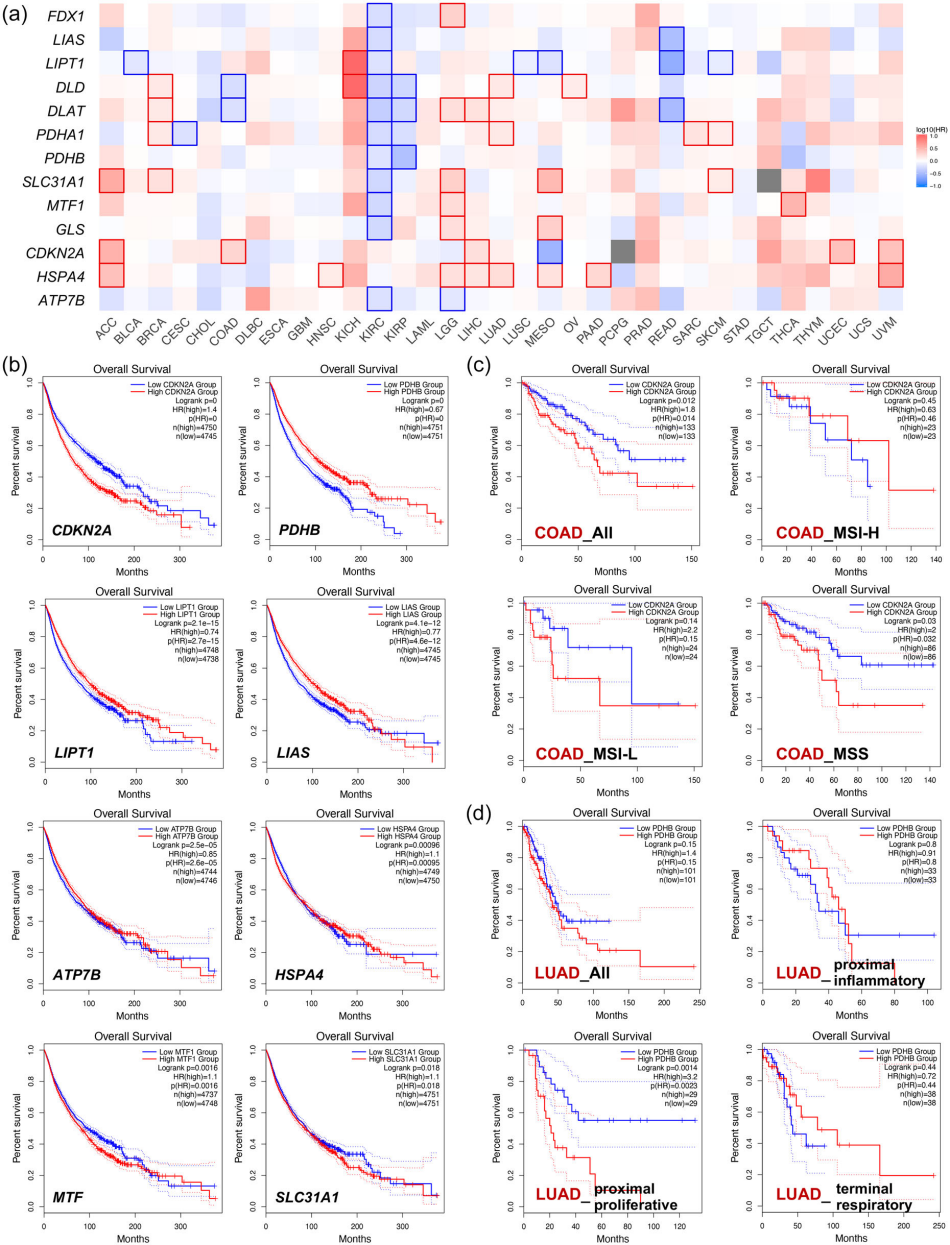
Correlation between CRG expression and overall survival of patients in different cancers. (a) A survival map (OS) of 33 cancers was obtained using the online tool GEPIA2. (b) Kaplan–Meier survival plots (OS) comparing high and low expression of different CRGs in pan‐cancer. (c) Kaplan–Meier survival plots (OS) comparing high and low expression of *CDKN2A* in all and each subtype of COAD patients. (d). Kaplan–Meier survival plots (OS) comparing high and low expression of *PDHB* in all and each subtype of LUAD patients.

Next, we compared the prognostic difference between the CRG high‐expression group and the low‐expression group to explore the predictive value of CRGs in human cancer. The overall survival (OS) map of 33 human cancers is shown in Figure 2a. We found a close relationship between CRG expression and most cancer types, especially KIRC, low grade glioma (LGG) and mesothelioma (MESO). The overall survival of pan‐cancer was also correlated with the expression of *CDKN2A*, *PDHB*, *LIPT1*, *LIAS*, *ATP7B*, *HSPA4*, *MTF* and *SLC31A1* (Figure 2b). It should be noted that even though a CRG was associated with the survival of certain cancers, the prognostic value was possibly insignificant on the subtype (Figure 2c and Supporting information, Figure S2). For example, high expression of *CDKN2A* suggested higher mortality for COAD, but not the subtype of MSI–high and MSI–low (MSI‐H and MSI‐L (Figure 2c). It was still interesting that the prognostic value was presented only in a certain subtype rather than in all the patients of a tumour. As shown in Figure 2d, *PDHB* expression was significantly associated with the OS of proximal proliferative LUAD rather than other subtypes, which reminded us that CRGs may have different effects on different subtypes of a tumour.

### 3.3 Genetic alteration of CRGs and prognostic value in human cancers

**FIGURE 3:**
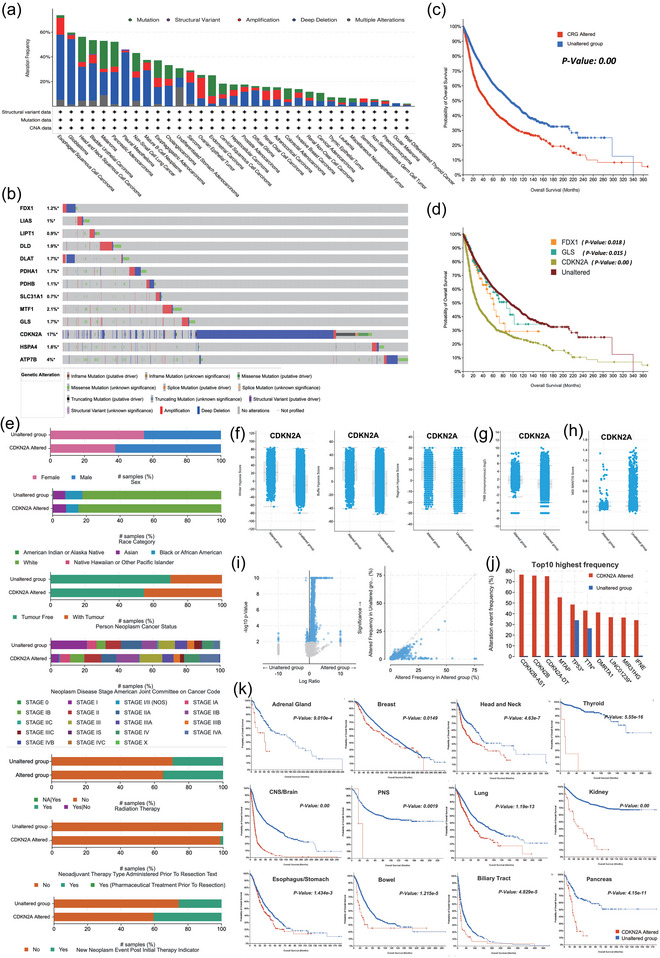
Feature and prognostic value of the genetic alteration of CRGs in different cancers. (a) Genetic alteration frequency and type of the CRGs in different cancers. (b) Alteration profile plot of each CRG in pan‐cancer. (c) Kaplan–Meier survival plots (OS) comparing CRG altered and unaltered group in pan‐cancer. (d) Kaplan–Meier survival plots (OS) comparing CRG‐altered patients and unaltered patients in pan‐cancer, and 3 significant CRGs including *FDX1*, *GLS* and *CDKN2A* were plotted. (e) The clinical features of *CDKN2A*‐altered individuals including sex, race, cancer status, neoplasm disease stage, therapy and new neoplasm event. (f–h) Comparison of hypoxia score (f), TMB (g), and MSI mantis score (h) between *CDKN2A*‐altered and unaltered group. (i) scatter plot of alteration co‐occurrence. (J) Top 10 frequent co‐alteration genes of *CDKN2A* in the samples. (k) Kaplan–Meier survival plots (OS) comparing *CDKN2A*‐altered and ‐unaltered group in the tumour of the adrenal gland, breast, head and neck, thyroid, central nervous system (CNS)/brain, peripheral nervous system (PNS), lung, kidney, oesophagus/stomach, bowel, biliary tract and pancreas.

The genetic alteration of CRGs in pan‐cancer was explored using the online tool cBioPortal. As shown in Figure 3a, CRG alteration was most pronounced in Esophageal Squamous Cell Carcinoma (ESCC), and the type of genetic alteration in most tumours was gene deletion. Of all the 13 CRGs, CDKN2A was the most frequently altered gene. The alteration rate could be as high as 17% in the 10953 tumour patients (Figure 3b), Deep deletion was the major alteration type of CDKN2A, and ESCC was also the most frequently altered tumour type (Figure S3). The overall survival was compared between CRG‐altered and unaltered patients at the pan‐cancer level. We found that the OS of the altered group was significantly lower than the unaltered group, which suggested that CRG alteration was associated with poor prognosis for cancer patients (Figure 3c). The overall survival was further compared between each CRG‐altered patients and unaltered ones to further disclose the prognostic value of each CRG alteration. The result indicated that three CRGs, *FDX1*, *GLS* and *CDKN2A*, were significantly associated with patient survival (Figure 3d). Specifically, we explored the clinical characteristics of *CDKN2A*‐altered individuals including sex, race, cancer status, neoplasm disease stage, therapy and new neoplasm event (Figure 3e). We found that there were more male and white people in the *CDKN2A*‐altered population. More tumour‐bearing individuals were in the *CDKN2A*‐altered group with a higher tumour stage. More patients in the *CDKN2A*‐altered group needed radiation and neoadjuvant therapy and experienced new neoplasm events. In addition, *CDKN2A*‐altered tumour had a higher hypoxia score (Figure 3f) and TMB (Figure 3g), but a lower MSI mantis score (Figure 3h). Alteration co‐occurrence was more frequent in *CDKN2A*‐altered samples compared with unaltered ones (Figure 3i). Genes such as *MTAP* (55.26%), *TP53* (48.60%), *TTN* (42.97%), and *DMRTA1* (41.17%) were usually co‐altered with *CDKN2A* in the samples (Figure 3j). Significantly, we found *CDKN2A* alteration was related to lower survival in the tumour of the adrenal gland, breast, head, neck, thyroid, central nervous system (CNS)/brain, peripheral nervous system (PNS), lung, kidney, oesophagus/stomach, bowel, biliary tract and pancreas (Figure 3k). These findings suggested that CRG alteration was closely correlated with poor prognosis, especially *CDKN2A* alteration.

### 3.4 Methylation and CNV of the CRGs in different cancers

**FIGURE 4:**
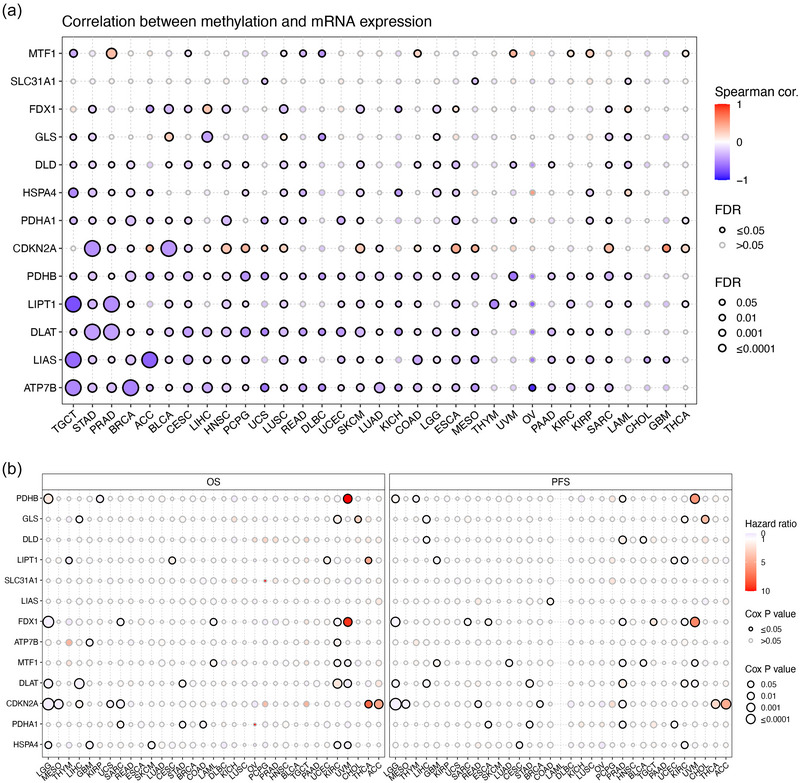
The methylation feature of CRGs in pan‐cancer. (a) The correlation between methylation and mRNA level of each gene. (b) The correlation between CRG methylation and OS/PFS of tumour patients.

Further, we explored the methylation characteristic of CRGs in pan‐cancer. Results indicated that the methylation levels of CRGs were generally negatively correlated with mRNA levels (Figure 4a). Subsequently, the correlation between CRG methylation and patient survival was also shown in Figure 4b.

**FIGURE 5:**
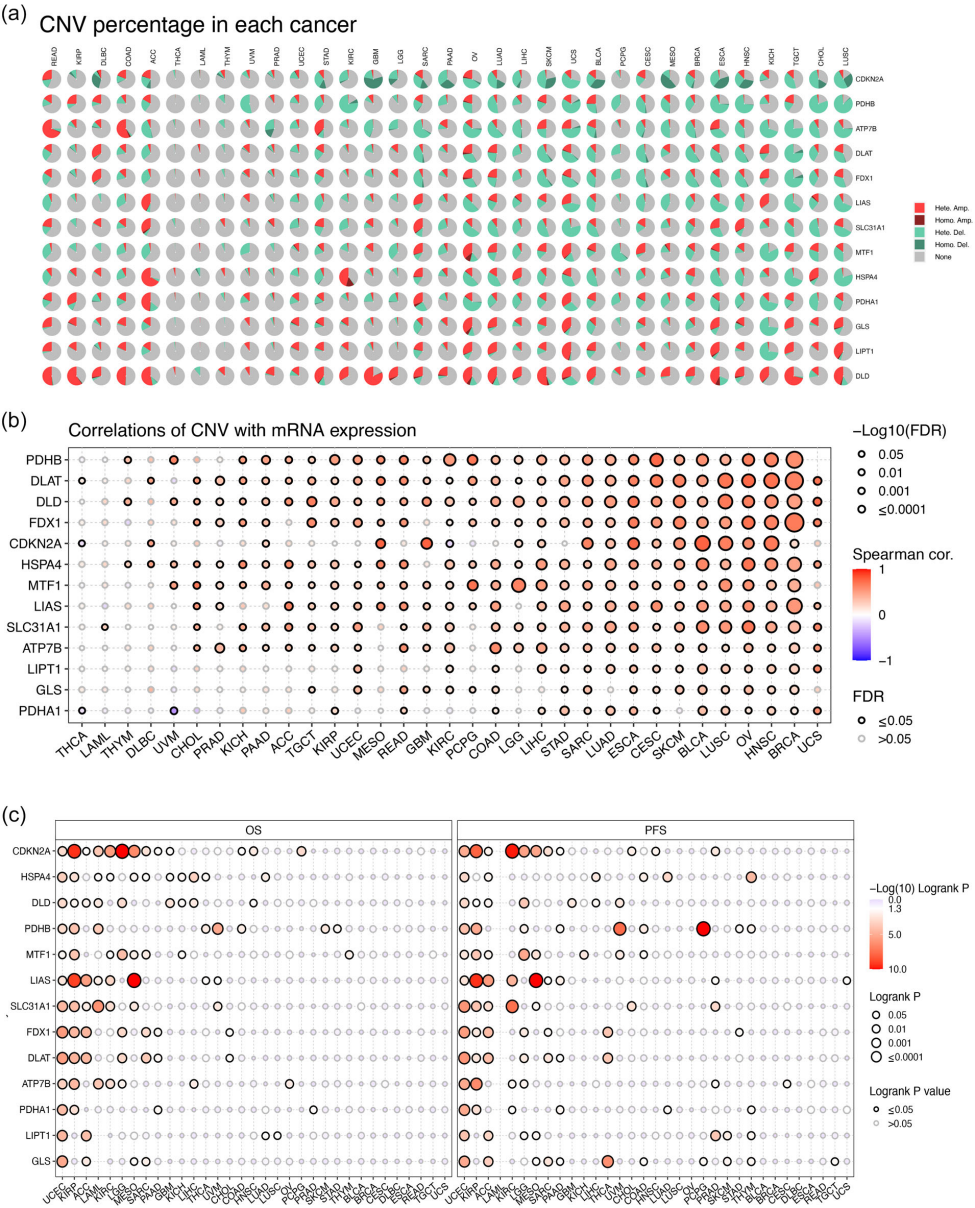
The CNV of CRGs in pan‐cancer. (a) Pie plot summarizing the CNV of CRGs in different tumour types. (b) The correlation between CNV and gene expression. (c) The correlation between the CVN level of each CRG and patient survival in pan‐cancer.

The proportion of different types of CNV (including heterozygous amplification, heterozygous deletion, homozygous amplification and homozygous deletion) of each CRG in pan‐cancer is shown in Figure 5a. The CNV levels of most CRGs were positively correlated with their mRNA levels (Figure 5b). Multiple significant correlations between the CVN level of each CRG and patient survival are demonstrated in Figure 5c.

### 3.5 Relationship between CRG score and the immune cell infiltration in pan‐cancer

**FIGURE 6:**
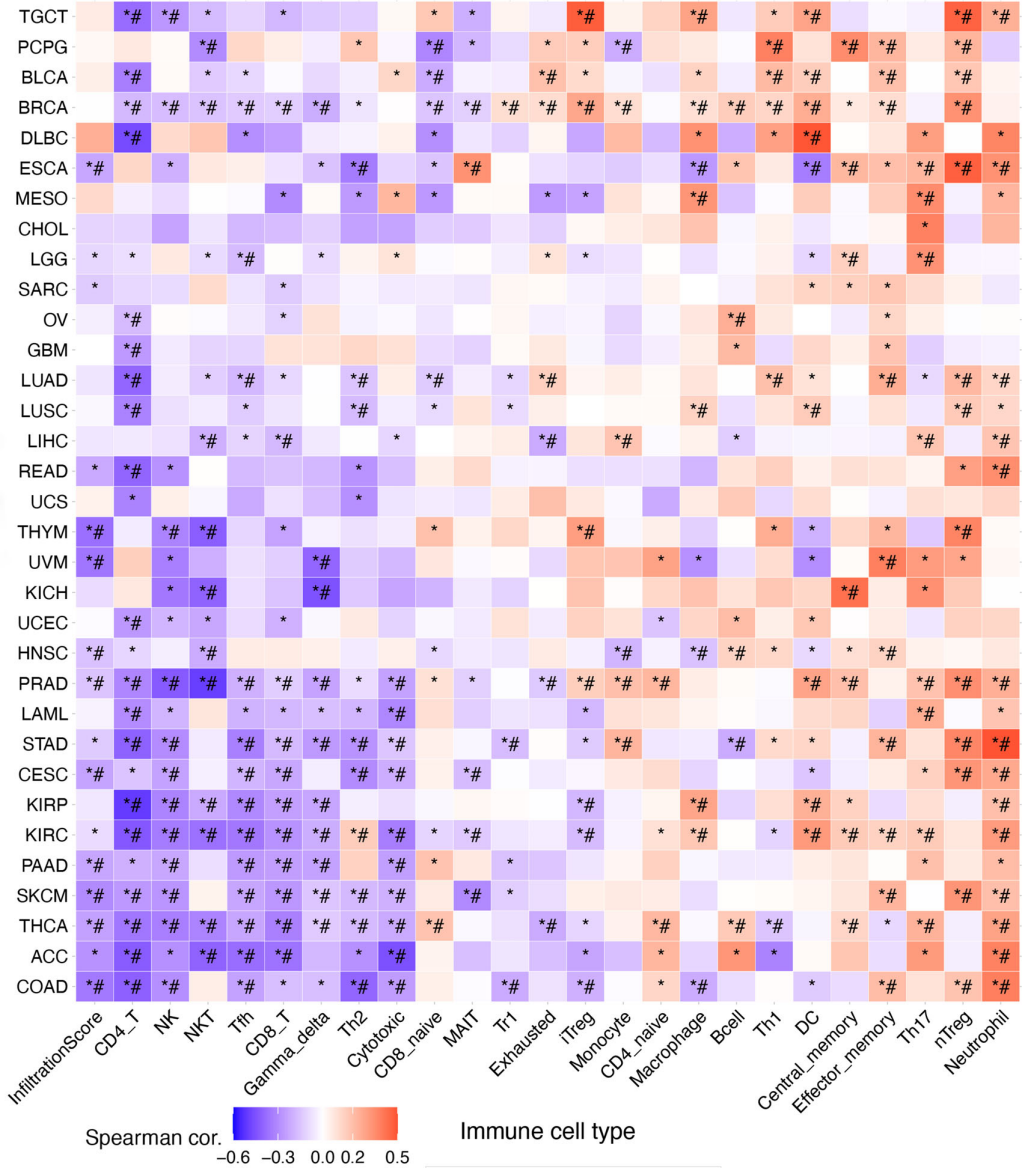
The correlation between CRG score and immune cell infiltration based on ImmuCellAI. ^*^ *P* ≤ 0.05; ^#^false discovery rate (FDR) ≤ 0.05.

It is known that the tumour microenvironment (TME) impacts the cancer prognosis, and thus we explored the relationship between CRGs and immune cell infiltration in tumours. Based on the ImmuCellAI database, we found that at the pan‐cancer level, the CRG score is highly correlated with most immune cells (Figure 6). A negative correlation between CRG and infiltration score was present in the majority of the cancers. The CRG score was negatively correlated with the infiltration of immune cells including CD4^+^T cells, NK, Tfh, CD8^+^T, γδT and Th2 cells, but positively connected to the infiltration of neutrophil, nTreg, and Th17 cells in most cancers.

### 3.6 Correlations between *CDKN2A* expression and immune regulation

**FIGURE 7:**
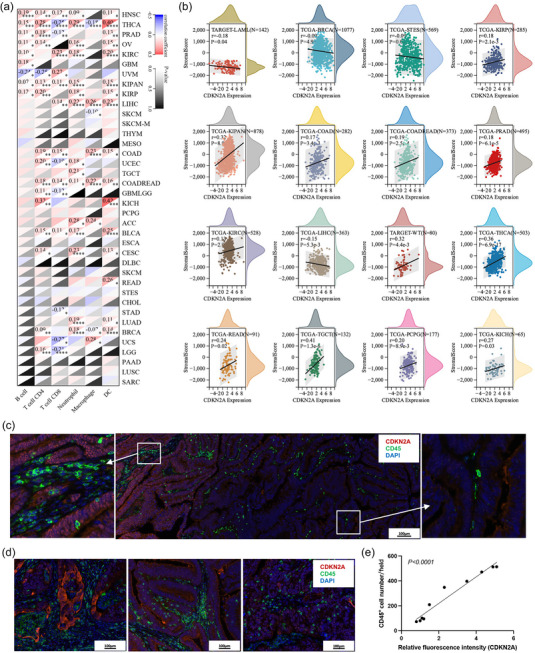
The correlation between *CDKN2A* expression and immune cell infiltration. (a) Correlation analysis between *CDKN2A* expression and the infiltration score of immune cells including B cells, CD4^+^ T cells, CD8^+^ T cells, neutrophils, macrophages and dendritic cells in different cancers. (b) The correlation between *CDKN2A* expression and stromal score in different cancers. (c) Fluorescence images of colon cancer staining CDKN2A (red) and CD45 (green). (d) Fluorescence images of colon cancer from different patients staining CDKN2A (red) and CD45 (green). (e) statistical analysis of the correlation between CDKN2A expression and CD45^+^ immune cells. ^*^ *P* ≤ 0.05, ^**^ *P* ≤ 0.01, ^***^ *P* ≤ 0.001, ^****^ *P* ≤ 0.0001.

Given the significant value of *CDKN2A* expression and alteration for cancer survival, we further evaluate the correlation between *CDKN2A* expression and immune cell infiltration. The correlation analysis was performed between *CDKN2A* expression and the infiltration score of immune cells including B cells, CD4^+^ T cells, CD8^+^ T cells, neutrophils, macrophages and dendritic cells in different cancers, and positive correlations were found in most cancers (Figure 7a). Moreover, the stromal score for immune cell infiltration was calculated, and we found significant correlations between *CDKN2A* expression and stromal score in 16 cancers (Figure 7b). Consistently, positive correlations were demonstrated in most cancer types including KIRP, the pan‐kidney cohort (KIPAN), COAD, colorectal adenocarcinoma/rectal adenocarcinoma (COADREAD), PRAD, KIRC, high‐risk Wilms tumour (TARGET‐WT), THCA, rectum adenocarcinoma (READ), testicular germ cell tumours (TGCT), pheochromocytoma and paraganglioma (PCPG) and KICH. To further verify the correlation between *CDKN2A* expression and immune cell infiltration, fluorescence staining of colon cancer with CDKN2A (red) and CD45 (green) was performed. We found that areas with stronger fluorescence staining of CDKN2A had more immune cell infiltration (Figure 7c). Consistently, fluorescence images of colon cancer from different patients demonstrated a positive correlation between CDKN2A expression and the number of CD45^+^ immune cells (Figure 7d, e).

Moreover, we evaluated the correlation between the expression of *CDKN2A* and 150 marker genes of immunomodulators (including 41 chemokines, 18 receptors, 21 MHCs, 24 immune inhibitors, and 46 immune stimulators) (Supporting information, Figure S4). The result indicated that positive correlations existed in most cancers, except for acute myeloid leukemia (LAML), acute lymphoblastic leukemia (ALL), uterine carcinosarcoma (UCS), stomach adenocarcinoma (STAD), squamous cell carcinoma of the oesophagus (STES), GBM, skin cutaneous melanoma (SKCM), cholangiocarcinoma (CHOL), DLBC and ESCA.

### 3.7 Correlations between *CDKN2A* and immune checkpoint in pan‐cancer

The correlations between the expression of *CDKN2A* and 60 immune checkpoint genes (including 24 inhibitory and 36 stimulatory genes) were also evaluated. As shown in Supporting information, Figure S5, The positive correlation between *CDKN2A* and the immune checkpoint genes was generally shown in cancers, especially ovarian serous cystadenocarcinoma (OV), KICH, KIPAN, and TGCT. However, most correlations were not significant in the cancers like MESO, uterine corpus endometrial carcinoma (UCEC), STAD, STES, THYM, LUSC, glioblastoma multiforme, brain lower‐grade glioma (GBMLGG), LGG, LAML, UCS, DLBC, CHOL, GBM, SKCM and ALL.

### 3.8 Correlations between CDKN2A and genomic heterogeneity in different tumours

As shown in Supporting information, Figure S6, the correlations between *CDKN2A* expression and TMB of different tumours were investigated and we found a significant correlation in 12 tumours, including eight positive correlations (GBMLGG, *P* = 0.0310; LUAD, *P* = 0.0002; KIPAN, *P* = 0.0371; LIHC, *P* = 0.0123; THCA, *P* = 0.0222; BLCA, *P* = 0.0021; adrenocortical carcinoma (ACC), *P* = 0.0193; KICH, *P* = 0.0125) and four negative correlations (cervical squamous cell carcinoma and endocervical adenocarcinoma (CESC), *P* = 0.0005; STES, *P* = 0.0089; STAD, *P* = 0.0070; UCEC, *P* = 5.4706e‐7). There were 15 significant correlations between *CDKN2A* expression and MSI, including nine positive correlations (GBM, *P* = 0.0251; GBMLGG, *P* = 0.0330; BRCA, *P* = 0.00006; PRAD, *P* = 0.0117; KIRC, *P* = 0.0125; THCA, *P* = 0.0317; READ, *P* = 0.0312; BLCA, *P* = 0.0434; KICH, *P* = 0.0114) and six negative correlations (CESC, *P* = 0.0002; STES, *P* = 0.0006; STAD, *P* = 0.0006; UCEC, *P* = 1.3796e‐7; OV, *P* = 0.0376; UCS, *P* = 0.0189). The NEO score in eight tumours was correlated with *CDKN2A* expression, including four positive correlations (GBMLGG, *P* = 0.0470; LGG, *P* = 0.0131; READ, *P* = 0.0145; BLCA, *P* = 0.0125) and four negative correlations (CESC, *P* = 0.00002; UCEC, *P* = 0.000016; UCS, *P* = 0.0034; CHOL, *P* = 0.0315). Eighteen significant correlations between *CDKN2A* expression and HRD were identified, including nine positive correlations (GBMLGG, *P* = 0.0077; LGG, *P* = 0.0032; LUAD, *P* = 0.0003; COAD, *P* = 0.0007; COADREAD, *P* = 0.0062; BRCA, *P* = 9.4061e‐31; STES, *P* = 1.0605e‐7; KIPAN, *P* = 1.3189e‐8; STAD, *P* = 5.2830e‐13; PRAD, *P* = 0.0000017; UCEC, *P* = 4.0026e‐11; KIRC, *P* = 4.9121e‐9; LIHC, *P* = 2.9420e‐7; BLCA, *P* = 3.0697e‐9; ACC, *P* = 0.0047; KICH, *P* = 0.0034) and two negative correlations (HNSC, *P* = 0.0041; MESO, *P* = 0.0002).

### 3.9 Correlations between *CDKN2A* expression and stemness scores in different tumours

The correlations between *CDKN2A* expression and the stemness scores in different tumours were further evaluated in Supporting information, Figure S7, and 18 significant correlations were identified using RNAss score, including 10 positive correlations (GBM, *P* = 0.0015; CESC, *P* = 0.0245; LUAD, *P* = 5.7563e‐7; BRCA, *P* = 3.3925e‐17; UCEC, *P* = 0.0245; HNSC, *P* = 0.0059; LIHC, *P* = 1.4935e‐9; OV, *P* = 0.0011; SKCM, *P* = 0.0412; BLCA, *P* = 0.0084) and eight negative correlations (COAD, *P* = 0.0107; COADREAD, *P* = 0.0015; KIRP, *P* = 0.00002; KIPAN, *P* = 3.2352e‐17; KIRC, *P* = 0.0008; THCA, *P* = 1.5282e‐12; READ, *P* = 0.0424; TGCT, *P* = 1.4810e‐11). There were eight significant correlations between *CDKN2A* expression and EREG.EXPss score, including two positive correlations (BRCA, *P* = 0.0001; PRAD, *P* = 1.4493e‐11) and six negative correlations (LGG, *P* = 0.0145; COADREAD, *P* = 0.03478; KIRP, *P* = 0.000029; HNSC, *P* = 0.0041; TGCT, *P* = 1.8539e‐7; SKCM, *P* = 0.0182). Eight positive correlations (LAML, *P* = 0.0065; BRCA, *P* = 0.0324; STES, *P* = 0.0008; KIPAN, *P* = 0.0070; STAD, *P* = 8.5198e‐8; KIRC, *P* = 0.00003; LIHC, *P* = 1.9125e‐8; THCA, *P* = 4.9352e‐10) and seven negative correlations (GBMLGG, *P* = 0.0267; SARC, *P* = 0.0022; KIRP, *P* = 0.0223; TGCT, *P* = 0.0020; PCPG, *P* = 0.0338; UCS, *P* = 0.0326; BLCA, *P* = 0.0028) were identified between *CDKN2A* expression and DNAss score. There were eight positive correlations (LAML, *P* = 0.0015; BRCA, *P* = 0.0001; STES, *P* = 0.00035; KIPAN, *P* = 0.0115; STAD, *P* = 5.5276e‐8; KIRC, *P* = 0.00002; LIHC, *P* = 5.4893e‐10; THCA, *P* = 4.0464e‐14) and six negative correlations (GBMLGG, *P* = 0.0435; SARC, *P* = 0.0025; TGCT, *P* = 0.0021; PCPG, *P* = 0.0130; UCS, *P* = 0.0301; BLCA, *P* = 0.0006) between *CDKN2A* expression and EREG‐METHss score. As for DMPss score, we found 10 significant correlations including five positive correlations (STES, *P* = 0.0087; STAD, *P* = 0.000002; KIRC, *P* = 0.00004; LIHC, *P* = 0.000006; THCA, *P* = 0.0007) and five negative correlations (CESC, *P* = 0.0005; SARC, *P* = 0.0061; KIRP, *P* = 0.0066; TGCT, *P* = 0.0006; UCS, *P* = 0.0167). Moreover, 14 significant correlations were found between *CDKN2A* expression and ENHss score, including seven positive correlations (LAML, *P* = 0.0351; STES, *P* = 0.0006; KIPAN, *P* = 0.0002; STAD, *P* = 3.3838e‐7; PRAD, *P* = 0.0398; KIRC, *P* = 0.0103; LIHC, *P* = 0.000001) and seven negative correlations (GBMLGG, *P* = 0.0098; CESC, *P* = 0.0232; SARC, *P* = 0.0007; KIRP, *P* = 0.0088; UCEC, *P* = 0.0283; TGCT, *P* = 0.0239; BLCA, *P* = 0.0039).

### 3.10 The correlation between CRG expression and drug sensitivity in pan‐cancer

**FIGURE 8:**
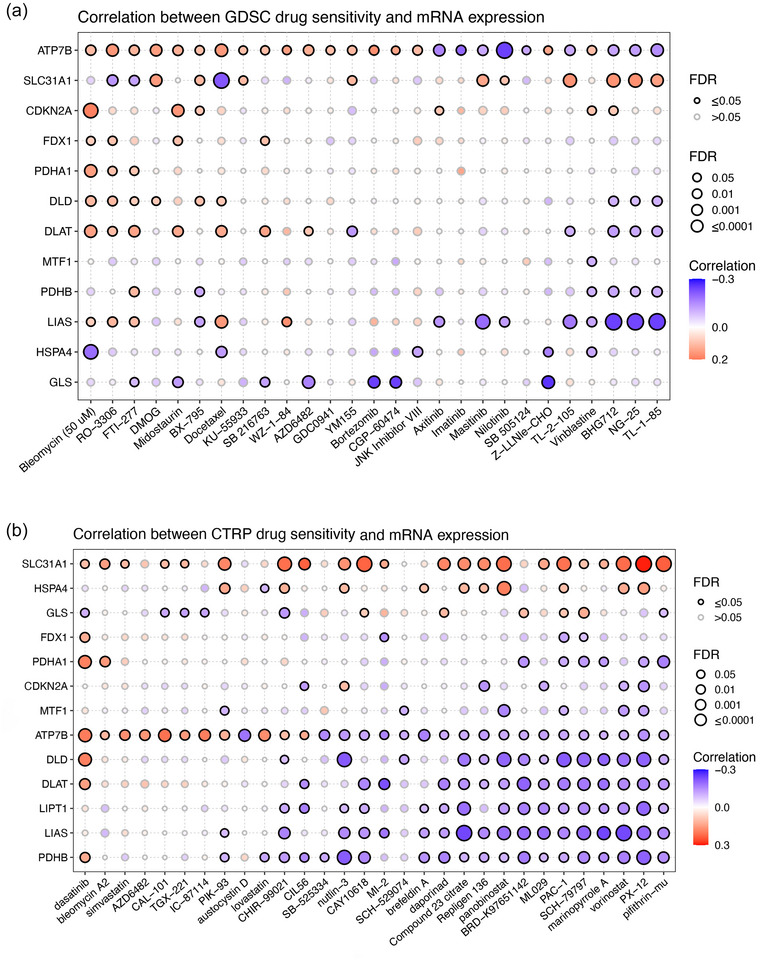
The correlation between CRG expression and drug sensitivity in pan‐cancer. (a) The correlation between CRG expression and GDSC drug IC_50_. (b) The correlation between CRG expression and CTRP drug IC_50_.

To further verify whether patients with different CRG expressions showed different sensitivity to chemotherapeutics, the correlation between CRG expression and drug IC_50_ in pan‐cancer was performed using GDSC and CTRP data. As shown in Figure 8, significant correlations were found between CRG and IC_50_ of multiple drugs. The expression of *ATP7B* was closely associated with all the 27 GDSC drugs (including 18 positive correlations and 9 negative correlations) (Figure 8a) and 30 CTRP drugs (including 11 positive correlations and 19 negative correlations) (Figure 8b).

## 4 DISCUSSION

Cancer is a multifactorial disease. Pathways including metabolism, cell death and immune response are known to participate in tumour progression. Effective therapeutic approaches are still limited and cuproptosis investigation may bring new hope for cancer treatment. In the present research, a comprehensive bioinformatics investigation was performed to systematically analyse the expression profile, methylation, and CNV of 13 CRGs and further explore their prognostic significance and their relationship to TIME and drug resistance in pan‐cancer. Particularly, we found that *CDKN2A* was closely correlated to pan‐cancer prognosis and further identified its clinical features as well as correlations with TIME alteration, genomic heterogeneity and tumour stemness.

Pyruvate dehydrogenase E1 subunit beta (PDHB) is part of the pyruvate dehydrogenase complex that was found to catalyse glucose derived pyruvate to acetyl‐CoA (Saunier et al., 2016) and thus regulate the key step from glycolysis to the TCA cycle in glucose metabolism. Previous studies demonstrated that PDHB was involved in cell growth, invasion and metabolism in colorectal cancer (Zhu et al., 2017). In our study, we found that the expression of CRGs was significantly correlated with pathological stages, especially *PDHB*. Consistent with our results in Figure 1c, the expression of *PDHB* was identified as an independent prognostic marker that correlates with favourable clinical outcomes in gastric cancer (Sun et al., 2015).

Another important CRG, *CDKN2A* is involved in cell cycle regulation and the p53 signalling pathway (Kreuger et al., 2023), which implies a vital role of CDKN2A in cancer development. Here we found that *CDKN2A* was widely overexpressed in different tumours, and patients with high *CDKN2A* expression were closely associated with lower overall survival compared to those with low *CDKN2A* expression. It is consistent with the current finding that overexpressed *CDKN2A* was correlated with the poor prognosis in colorectal cancer (CRC) (Kang et al., 2022; Wang et al., 2022), LIHC (Luo et al., 2021) and bladder cancer (Worst et al., 2018). These results suggested the potential of *CDKN2A* as an original prognostic predictor for cancers. However, it should be noted that even though a CRG is associated with the survival of certain cancers, the prognostic value is possibly insignificant for the subtype. As shown in our study, high expression of *CDKN2A* suggested higher mortality for COAD, but not the subtype of MSI‐H and MSI‐L (Figure 2c). In addition, the prognostic value was presented only in a certain subtype rather than in all the patients of a tumour, which reminded us that CRGs may have different effects on different subtypes of a tumour. Previous findings showed a close relationship between *CDKN2A* mutation and tumour mutation burden (Deneka et al., 2022) and it was found that the presence of *CDKN2A* deletion might induce the progression of ESCC (Liu et al., 2022). Similar to the above findings, deep deletion is presented in most cancer types. Of all these 13 CRGs, *CDKN2A* was the most frequently altered gene with an alteration rate as high as 17% in the 10,953 tumour patients, and the alteration of *CDKN2A* was significantly associated with poor prognosis in tumour of the adrenal gland, breast, head and neck, thyroid, CNS/brain, PNS, lung, kidney, oesophagus/stomach, bowel, biliary tract, and pancreas. Considering that high cuproptosis activity was associated with a favourable prognosis in multiple tumours, it is reasonable to assume that CDKN2A may lead to the poor prognosis of these tumours by negatively regulating the cuproptosis pathway. However, an in‐depth study is still required in the future to illustrate the mechanism.

Recent studies highlighted the indispensable and integral roles of the TME in cancer progression (Hinshaw & Shevde, 2019; Nakamura & Smyth, 2020). Immune infiltrates in the TME contribute to tumour development and affect the clinical outcomes of cancer patients. Existing findings have suggested that oxidative phosphorylation and TCA cycle metabolism are also crucial in immune cell activation and metabolism. Thus, immune cells may be equally sensitive to cuproptosis. In our study, we found that high cuproptosis activity tends to imply lower immune response and immune cell infiltration. CDKN2A was shown as an important negative regulator of cuproptosis (Tsvetkov et al., 2022). Thus, in our in‐depth analysis, positive correlations between *CDKN2A* expression and the immune cell modulation were shown in most cancers. As the most abundant immune cells residing in the TME, macrophages play vital roles in immune homeostasis (Hinshaw & Shevde, 2019). Tumour‐associated macrophages (TAMs) were even considered to be associated with higher mortality in cancer patients and could serve as a potential therapeutic target (Cassetta & Pollard, 2018; DeNardo & Ruffell, 2019). The present study demonstrated a positive correlation between *CDKN2A* expression and immune cell burden in colon cancer. Consistent with our findings, a recent study also confirmed a positive correlation between *CDKN2A* expression and macrophage infiltration in LIHC (Luo et al., 2021). The strong association between CRGs and immune regulation in most tumours suggested that cuproptosis may contribute to tumour microenvironment remodelling. Further study is still needed to fully disclose the role of CDKN2A in other cancers.

Immunotherapy, such as immune checkpoint blockade (ICB) therapy, has delivered promising clinical outcomes in multifarious cancers (Zhang & Zhang, 2020). Emerging evidence indicates the role of cuproptosis in remodelling the TME, enhancing immune cell infiltration and converting ‘cold tumours’ into ‘hot tumours’ that respond better to immunotherapy. Our study found positive correlations between CDKN2A and the immune checkpoint genes in cancers, especially OV, KICH, KIPAN and TGCT. Genomic heterogeneity is involved in therapy resistance of multiple cancers and can be used as immunotherapy biomarkers (Palmeri et al., 2022). Our study demonstrated close correlations between the expression of *CDKN2A* and genomic heterogeneity in different cancers, which suggested that CDKN2A may be a promising target for cancer treatment.

Cancer stemness, defined as the self‐renewal and tumour‐initiation potential of cancer stem cells (CSCs), is a cancer biology property featuring activation of CSC signalling networks. High stemness is usually associated with cancer distant metastasis and multitherapy resistance and leads to poor prognosis (Fabregat et al., 2016; Ge et al., 2017). The present study calculated the stemness scores according to a previous study (Malta, Colaprico et al., 2018), and found close correlations between *CDKN2A* expression and stemness scores. Such correlation varies in different tumours. Alterations in cancer genomes strongly influence clinical responses to treatment and in many instances are potent biomarkers for response to drugs. We also found that CRGs were associated with the sensitivity of multiple chemotherapeutics, which suggested that therapies targeting CRGs might improve drug sensitivity. The implications of copper‐based drugs in cancer therapy are multifaceted, including the emerging concept of ‘metalloallostery’, whereby dynamic copper binding at non‐catalytic sites regulates protein activity (Pham & Chang, 2023). Copper‐based therapies have the potential to improve cancer treatment by offering more targeted, personalized and effective approaches.

### 4.1 Limitations

Our bioinformatics study focused on copper‐related gene signatures offers substantial potential for advancing the understanding of cancer biology. Nevertheless, there are some limitations in the present study. First, although we performed immunohistochemistry using pathological sections to verify the bioinformatics results, further work should include an independent dataset and experimental validation done for all the relevant CRGs (such as qPCR, western blotting, or in vitro assays if applicable) to confirm the key findings. Second, the present study had not yet explored the potential mechanisms of CRGs in cancers. How CRGs affect immune infiltration and tumour progression needs to be fully illustrated in in vivo and in vitro experiments. In addition, we found different roles of CRGs (such as *CDKN2A*) as tumour suppressors or oncogenes in different cancers, which might be attributed to the diverse origins of cancers and tumour heterogeneity.

### 4.2 Conclusion

In conclusion, the present study systematically analysed the expression profile of 13 CRGs and further explored their prognostic significance and relationship to TIME and drug resistance in pan‐cancer. Particularly, the clinical features and correlations with TIME alteration, genomic heterogeneity and tumour stemness of CDKN2A were disclosed. Our findings provide additional evidence for cuproptosis‐based prognostic biomarkers and potential therapeutic targets for tumours.

## AUTHOR CONTRIBUTIONS

Yan Xu and Guohan Chen designed the study. Xinyu Ge, Tian Zhao, Zhengliang Sun and Chengbao Li analysed and interpreted the data. Xinyu Ge and Kaijing Wang wrote the initial draft of the paper. Jinyi Wang, Jie Liu, Zhengjun Chai, and Wen Zhang contributed to the discussion and revision. All authors have read and approved the final version of this manuscript and agree to be accountable for all aspects of the work in ensuring that questions related to the accuracy or integrity of any part of the work are appropriately investigated and resolved. All persons designated as authors qualify for authorship, and all those who qualify for authorship are listed.

## CONFLICT OF INTEREST

None declared.

## Supporting information



Table S1 and Figures S1–S7.

## Data Availability

All the relevant data can be found in the article and its Supplementary Material.
