# Supplementary material for: Clinical significance and immune microenvironment association of cuproptosis‐related genes in pan‐cancer
Source: Exp Physiol. 2025 Jun 25;111(2):539–55. doi: 10.1113/EP092310 (PMC12857452; doi:10.1113/EP092310)
Supplement: Supplementary file 1 — Table S1 and Figures S1–S7. [file EPH-111-539-s001.pdf]

Supplementary material

Clinical Significance and Immune Microenvironment Association of Cuproptosis Related Genes in Pan-Cancer

Xinyu Ge[1#], Kaijing Wang[2#], Tian Zhao[1#], Jinyi Wang[1], Jie Liu[1], Zhengliang Sun[1], Zhengjun Chai[1], Wen Zhang[1], Chengbao Li[4], Yan Xu[3*], Guohan Chen[1*]

1. Department of Thoracic Surgery, Shanghai East Hospital, Tongji University School of Medicine, Shanghai 200120, P.R. China;
2. Department of Hepatological Surgery, General Surgery, Shanghai East Hospital, Tongji University School of Medicine, Shanghai 200120, China;
3. Department of Pathology, Shanghai East Hospital, Tongji University School of Medicine, Shanghai 200120, China;
4. Department of Anesthesiology, Shandong Provincial Hospital Affiliated to Shandong First Medical University, Jinan, Shandong, 250021, China.

[#] Xinyu Ge, Kaijing Wang and Tian Zhao contributed equally to this work.

Running title: Landscape of CRGs in pan-cancer

[*] Address for Correspondence:

Yan Xu, MD

Shanghai East Hospital, Tongji University School of Medicine, No. 150 Jimo Rd., Shanghai 200120, China. Tel:0086-17368950025; Email: xuyan11230321@163.com;

Guohan Chen, MD, PhD

Shanghai East Hospital, Tongji University School of Medicine, No. 150 Jimo Rd., Shanghai 200120, China. Tel:0086-15316166057; Email: 1710459@tongji.edu.cn.

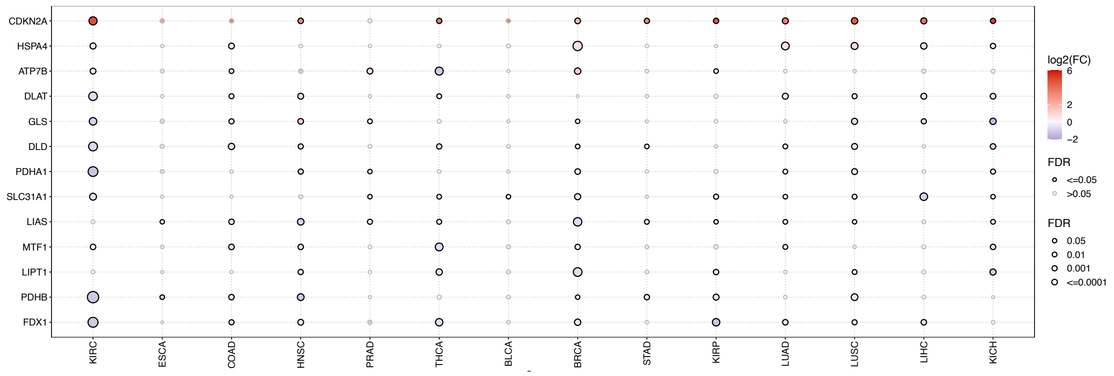

Supplementary Figure 1. Differential expression of 13 CRGs in various cancers. The expression of CRGs was compared between tumor tissue and normal tissue using GSCA platform. The cancer types which have more than 10 paired tumor and adjacent non-tumor samples, were selected to perform the differential analysis. The blue bubbles represent negative correlations and the red bubbles represent positive correlations. Bubble size was positively correlate with the FDR significance. Black outline border indicated FDR ≤ 0.05.

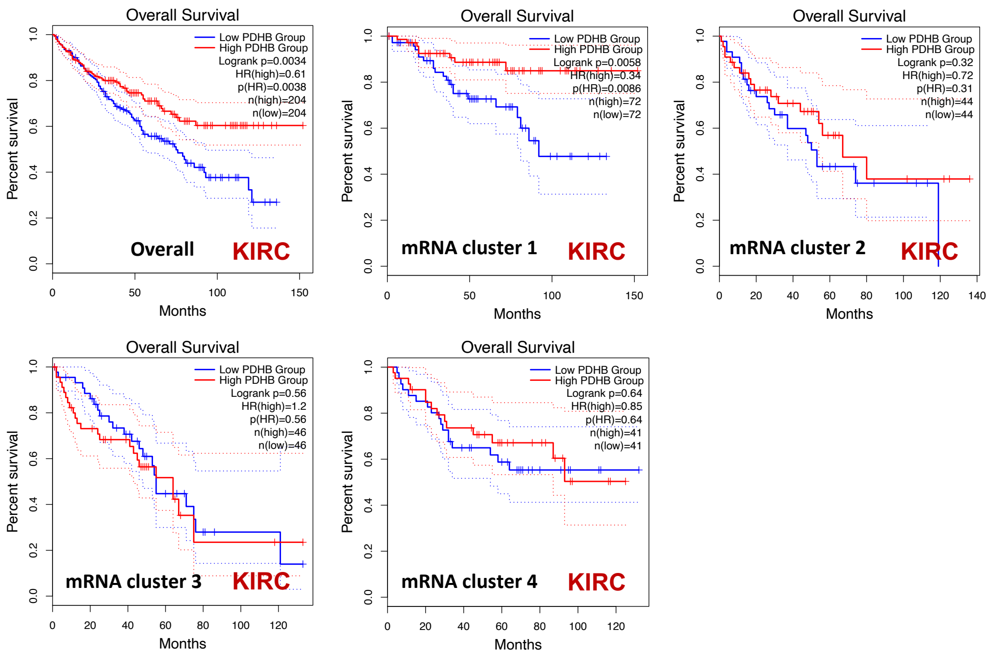

Supplementary Figure 2. The prognostic value of PDHB expression on overall and subtype of KIRC. Kaplan–Meier survival plots (OS) comparing high and low expression of PDHB in overall as well as each subtype of KIRC patients.

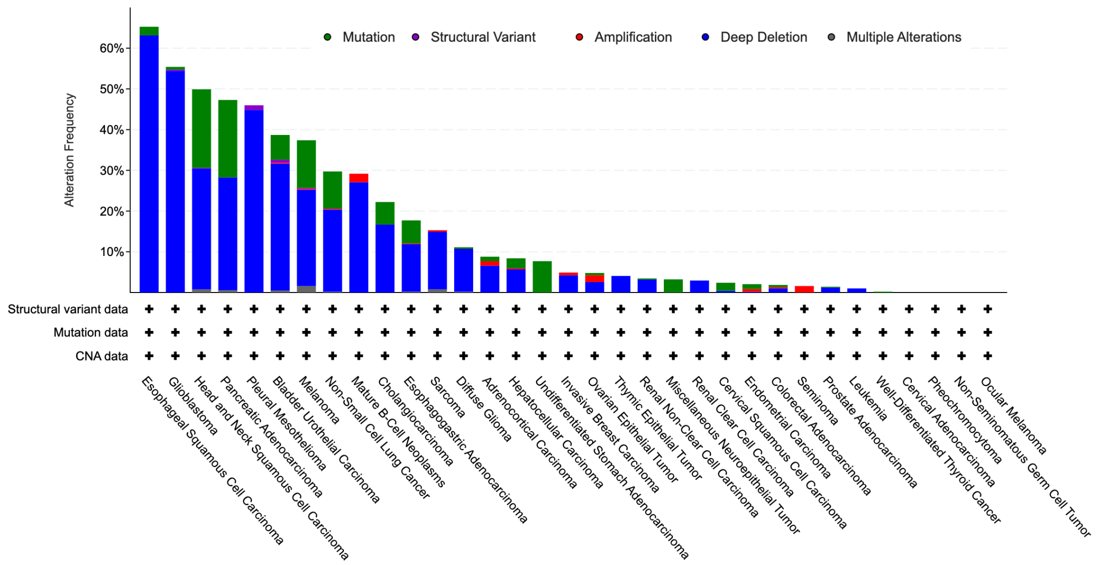

Supplementary Figure 3. Genetic alteration frequency and type of CDKN2A in different cancers.

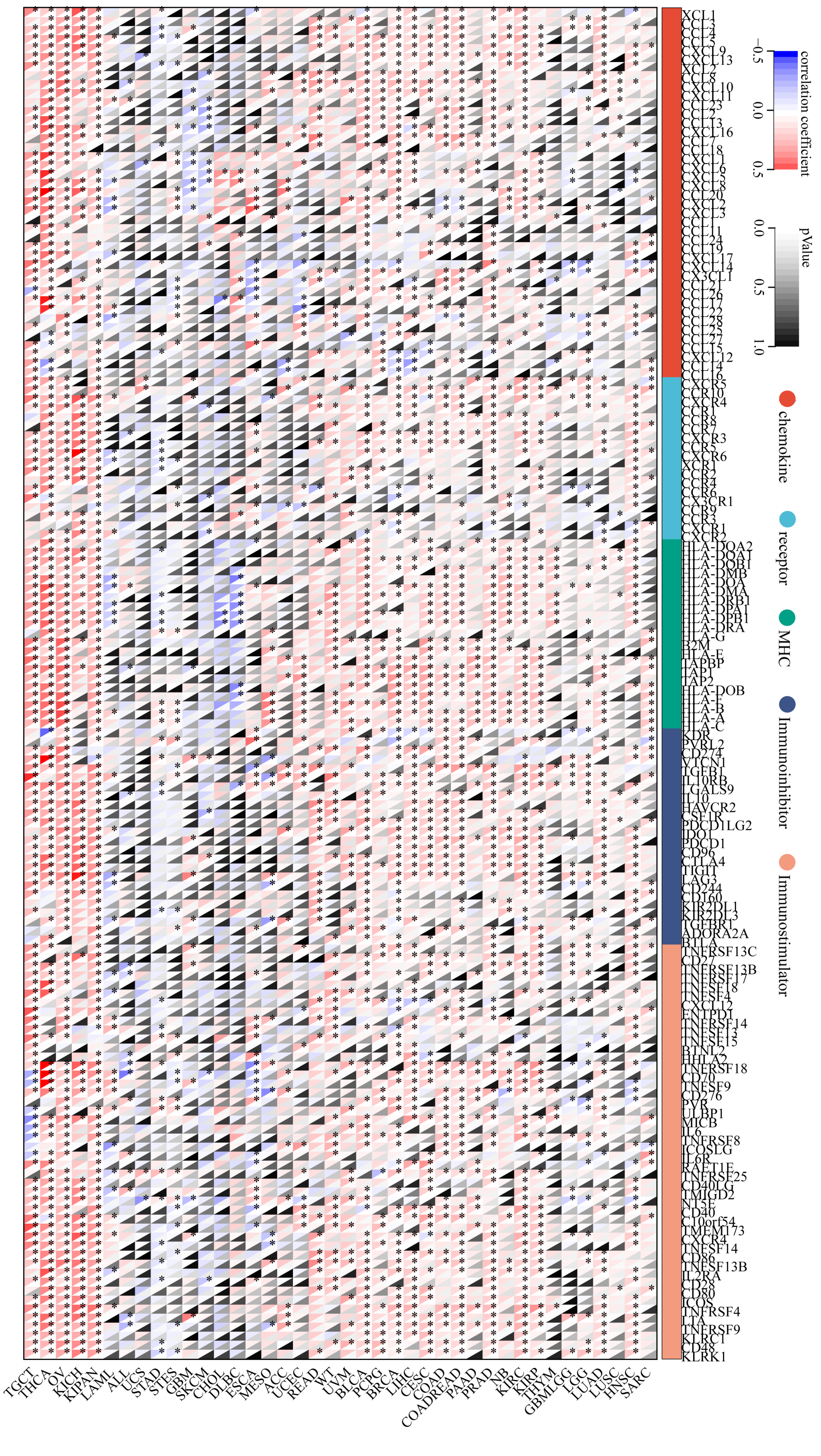

Supplementary Figure 4. The correlation between the expression of CDKN2A and immunomodulators in pan-cancer. *, P ≤ 0.05.

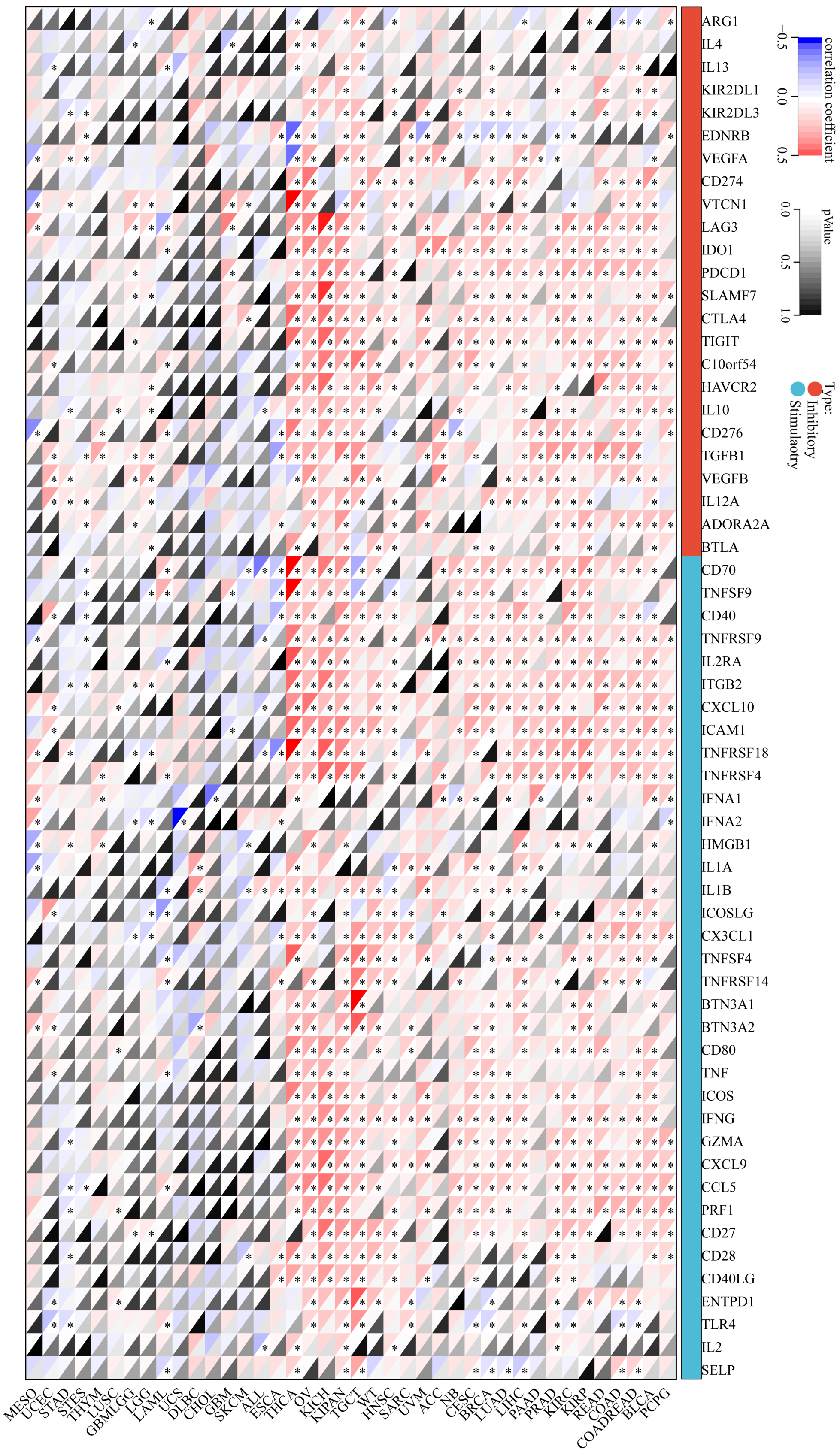

Supplementary Figure 5. The expression correlation between CDKN2A and immune checkpoint genes in pan-cancer. *, P ≤ 0.05.

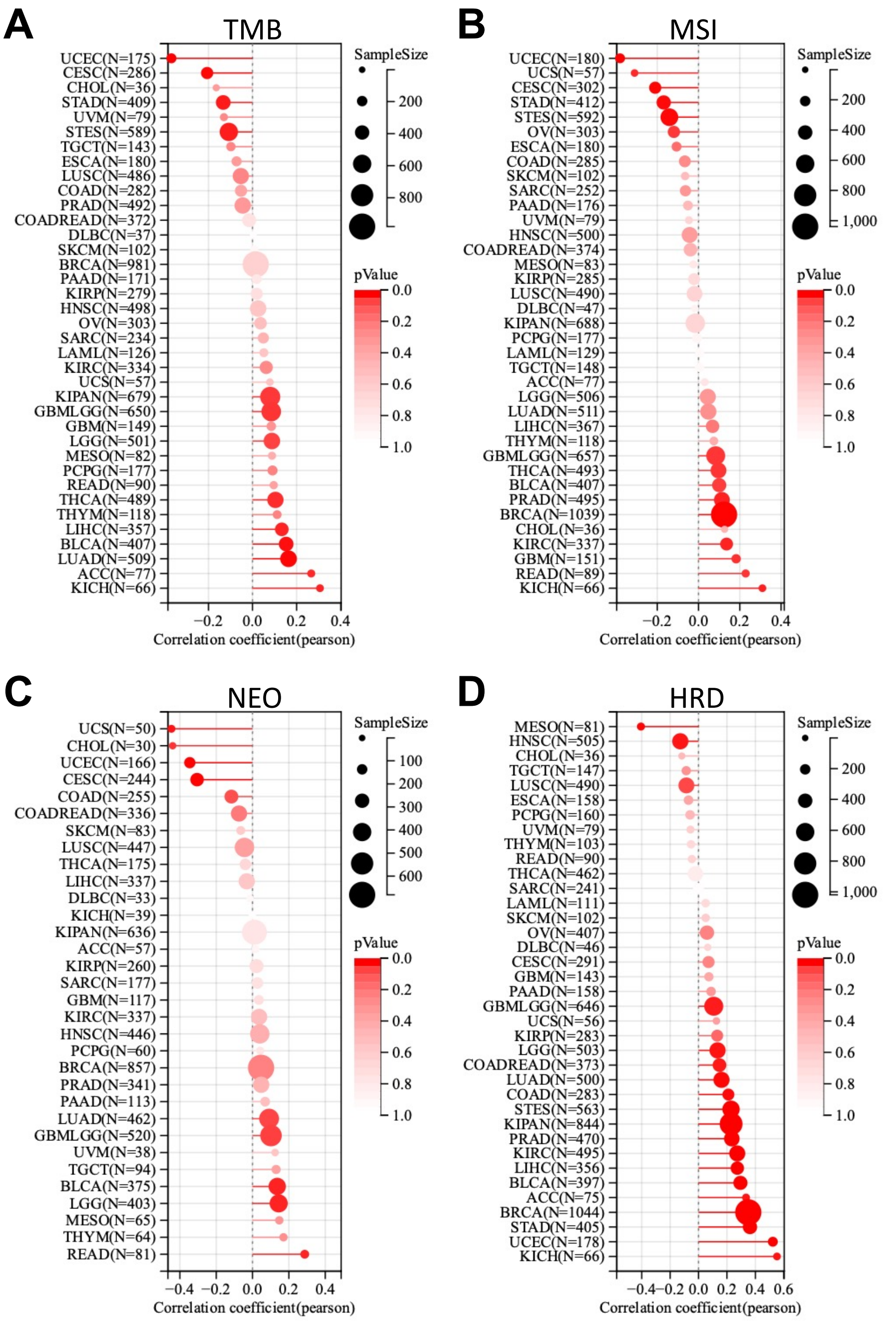

Supplementary Figure 6. The correlation between the expression of CDKN2A and genomic heterogeneity in pan-cancer, including (A) TMB, (B) MSI, (C) NEO, and (D) HRD.

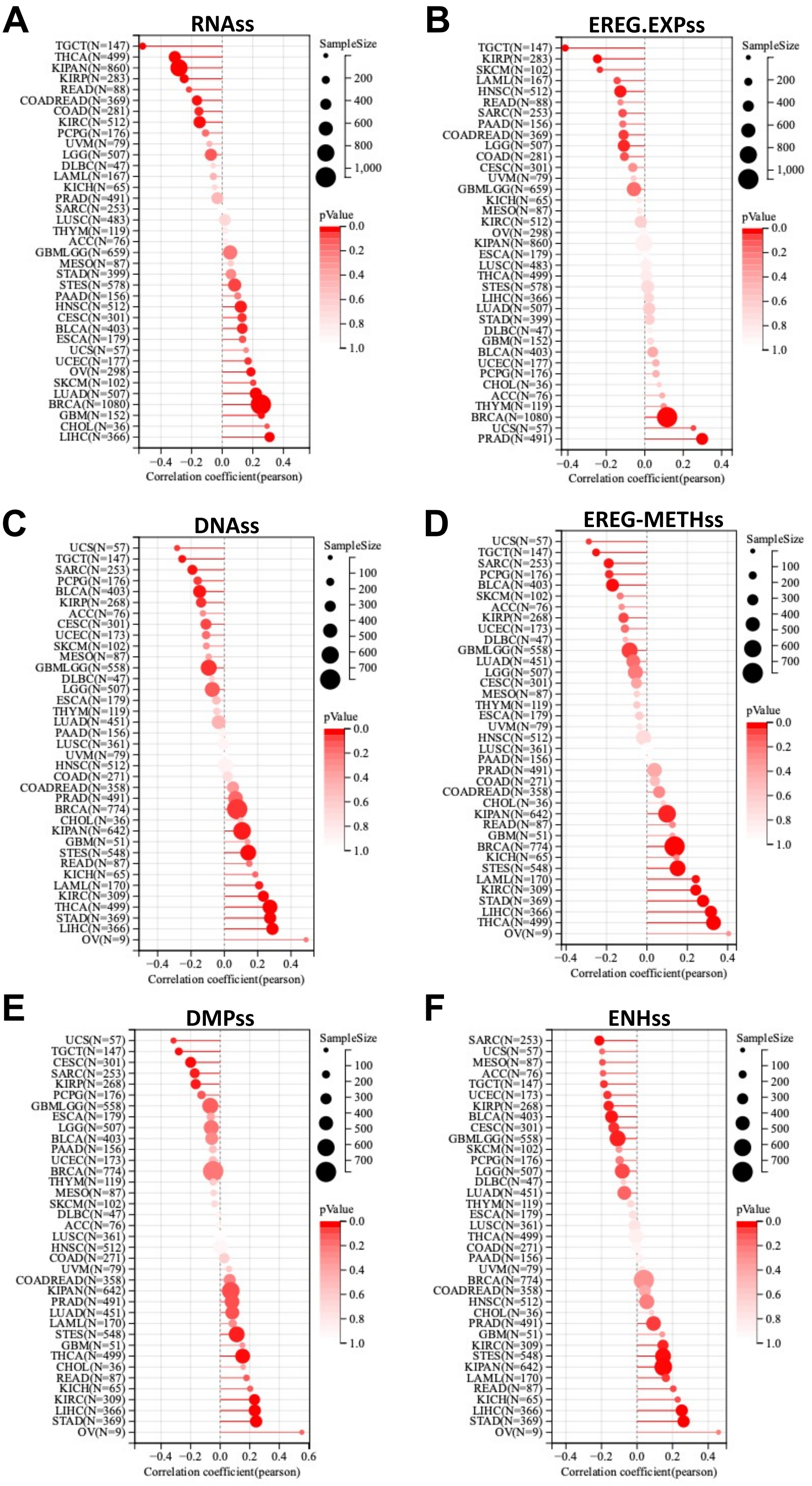

Supplementary Figure 7. The correlation between the expression of CDKN2A and 6 stemness scores in pan-cancer, including (A) RNAss, (B) EREG.EXPss, (C) DNAss, (D) EREG-METHss, (E) DMPss, and (F) ENHss.

Supplementary Table 1. The abbreviations and full names of all TCGA cancers

**Abbreviation**	**Full name**
ACC	Adrenocortical carcinoma
BLCA	Bladder Urothelial Carcinoma
BRCA	Breast invasive carcinoma
CESC	Cervical squamous cell carcinoma and endocervical adenocarcinoma
CHOL	Cholangiocarcinoma
COAD	Colon adenocarcinoma
COADREAD	Colon adenocarcinoma/Rectum adenocarcinoma Esophageal carcinoma
DLBC	Lymphoid Neoplasm Diffuse Large B-cell Lymphoma
ESCA	Esophageal carcinoma
FPPP	FFPE Pilot Phase II
GBM	Glioblastoma multiforme
GBMLGG	Glioma
HNSC	Head and Neck squamous cell carcinoma
KICH	Kidney Chromophobe
KIPAN	Pan-kidney cohort (KICH+KIRC+KIRP)
KIRC	Kidney renal clear cell carcinoma
KIRP	Kidney renal papillary cell carcinoma
LAML	Acute Myeloid Leukemia
LGG	Brain Lower Grade Glioma
LIHC	Liver hepatocellular carcinoma
LUAD	Lung adenocarcinoma
LUSC	Lung squamous cell carcinoma
MESO	Mesothelioma
OV	Ovarian serous cystadenocarcinoma
PAAD	Pancreatic adenocarcinoma
PCPG	Pheochromocytoma and Paraganglioma
PRAD	Prostate adenocarcinoma

READ	Rectum adenocarcinoma
SARC	Sarcoma
STAD	Stomach adenocarcinoma
SKCM	Skin Cutaneous Melanoma
STES	Stomach and Esophageal carcinoma
TGCT	Testicular Germ Cell Tumors
THCA	Thyroid carcinoma
THYM	Thymoma
UCEC	Uterine Corpus Endometrial Carcinoma
UCS	Uterine Carcinosarcoma
UVM	Uveal Melanoma
